# Cardiotoxicity of Fluoropyrimidines: Epidemiology, Mechanisms, Diagnosis, and Management

**DOI:** 10.3390/jcm10194426

**Published:** 2021-09-27

**Authors:** Michał Jurczyk, Magdalena Król, Aleksandra Midro, Magdalena Kurnik-Łucka, Adrian Poniatowski, Krzysztof Gil

**Affiliations:** Department of Pathophysiology, Jagiellonian University Medical College, 31-121 Krakow, Poland; mmagda.krol@student.uj.edu.pl (M.K.); aleksandra.midro@student.uj.edu.pl (A.M.); magdalena.kurnik@uj.edu.pl (M.K.-Ł.); adrian.poniatowski@uj.edu.pl (A.P.); krzysztof.m.gil@uj.edu.pl (K.G.)

**Keywords:** cancer treatment, chemotherapy, fluoropyrimidines, 5-fluorouracil, capecitabine, side effects, cardiotoxicity, heart failure, arrhythmias, vasospasm

## Abstract

Cancer is a growing public health problem; it is responsible annually for millions of deaths worldwide. Fluoropyrimidines are highly effective and commonly prescribed anti-neoplastic drugs used in a wide range of chemotherapy regimens against several types of malignancies. 5-fluorouracil and its prodrugs affect neoplastic cells in multiple ways by impairing their proliferation, principally through the inhibition of thymidylate synthase. Fluoropyrimidine-induced cardiotoxicity was described more than 50 years ago, but many details such as incidence, mechanisms, and treatment are unclear and remain disputed. Severe cardiotoxicity is not only life-threatening, but also leads to withdrawal from an optimal chemotherapy regimen and decreases survival rate. Differences in the frequency of cardiotoxicity are explained by different chemotherapy schedules, doses, criteria, and populations. Proposed pathophysiological mechanisms include coronary vasospasm, endothelial damage, oxidative stress, Krebs cycle disturbances, and toxic metabolites. Such varied pathophysiology of the cardiotoxicity phenomenon makes prevention and treatment more difficult. Cardiovascular disturbances, including chest pain, arrhythmias, and myocardial infarction, are among the most common side effects of this class of anti-neoplastic medication. This study aims to summarize the available data on fluoropyrimidine cardiotoxicity with respect to symptoms, incidence, metabolism, pathophysiological mechanism, diagnosis, management, and resistance.

## 1. History of 5-Fluorouracil (5-FU) Administration

The beginnings of cancer chemotherapy reach back to 1942 when Goodman et al. used nitrogen mustards (isolated from the poisonous gas, Yperite) to treat Hodgkin’s disease and leukemias [1]. In 1954, scientists revealed that liver tumors cells absorb more uracil than normal liver cells [2]. In 1957, Heidelberger et al. induced tumor cell death using 5-fluorouracil (5-FU), which became the first drug of the fluoropyrimidine class of chemotherapeutics [3] The FDA approved 5-FU in April 1962 to treat adenocarcinoma of the colon, rectum, breast, gastric, and pancreas. Yet, cardiac complications, among other adverse effects, began to be increasingly reported only a few years later.

Cardiotoxicity is a rare but serious complication of cytostatic agents, defined as a negative impact on heart function or cardiac cells. Nowadays, the definition includes not only clinical symptoms, but also changes in left ventricular ejection fraction or histopathological changes in cardiomyocytes [4,5]. Many studies identified anthracyclines as the most cardiotoxic chemotherapeutics; however, 5-FU and other fluoropyrimidines were also proven to affect cardiac cells and heart function [6,7,8]. Fluoropyrimidine cardiotoxicity was first described in 1969, and since then, many studies have confirmed these findings [2]. In 1990 Gradishar and Voke summarized that the incidence of FU-related ischemic-like symptoms fluctuated between 1.6%. and 10.2% [9,10,11].

Originally, one of the most significant routes of administration of 5-FU was a weekly bolus [12]. Seifert et al. conducted a prospective randomized trial that demonstrated the efficacy of 5-FU infusion [13]. De Gramont et al. proved that adding oxaliplatin to 5-FU could be beneficial in colon cancer treatment, creating the FOLFOX regimen, which remains the first-line treatment in metastatic colon cancer [14,15]. Nowadays, 5-FU is the third most used chemotherapeutic administered in many solid organ tumors, including adenocarcinomas of the gastrointestinal tract, and carcinomas of bladder, breast, head, and neck [7,16,17]. It is estimated that about 150,000 patients are potentially exposed to fluoropyrimidine treatment, annually [18].

Capecitabine is a prodrug of 5-FU. To form 5-FU, it undergoes hydrolysis in the liver and neoplastic tissues, where its activity is significantly higher. Capecitabine is used mainly in the therapy of gastrointestinal and breast cancers. The effectiveness of oral capecitabine in metastatic colorectal cancer is proven to be comparable to intravenous 5-FU while causing fewer adverse effects, such as diarrhea, stomatitis, neutropenia, and hair loss [19,20]. It is primarily recommended in monotherapy for patients who previously underwent surgery for III stage colorectal cancer [16]. The goal of this adjuvant therapy is to eradicate the micrometastases present at the time of surgery, thereby increasing the cure rate.

Through the years, cancer-associated survival-rate remarkably increased, and fluoropyrimidines remain the foundation of many antineoplastic treatments. Despite many studies explaining their mechanism of action (some of which are inconsistent with each other), there is still a lack of knowledge concerning their cardiotoxicity. The purpose of this study is to present an updated outline of fluoropyrimidine cardiotoxicity, namely its symptoms, incidence rate, metabolism, pathophysiological mechanisms, diagnosis, management, and resistance. Our results and discussion are integrated in each section.

PubMed was searched up through August 2020 for studies relating to fluoropyrimidine mechanisms published in English. We used the following terms: (cardiotoxicity, vasospasm, endothelial injury, or heart failure) and (5-fu, 5-fluorouracil, fluorouracil, capecitabine, or fluoropyrimidine). Three reviewers independently screened titles and abstracts, including appropriate studies about fluoropyrimidines. All disagreements in rating studies were resolved by discussion until a consensus was reached. Full-text articles were acquired based on study relevance; nevertheless, the classification procedure was not proved methodically. Original studies, case reports, and review articles were included. References of included studies were also checked for further appropriate articles.

## 2. Frequency of Fluoropyrimidine-Induced Cardiotoxicity

The frequency of symptomatic and asymptomatic cardiotoxicity during 5-FU treatment varies between studies. European Society of Cardiology (ESC) experts emphasized the possibility of underestimating myocardial damage during fluoropyrimidine treatment [21,22]. The American Society of Clinical Oncology remarked that heart diseases after cancer treatment are a growing health concern and that multidisciplinary teams should take care of these patients [23]. Nevertheless, the frequency of cardiotoxic events is disputable, ranging up to 68% [2,24]. This phenomenon is explained by different administration schedules, diagnostic criteria, screening examinations, included populations, and varying alertness to symptoms among patients. The ESC-estimated frequency of fluoropyrimidine induced myocardial ischemia is up to 10% in patients, depending on the route of administration, dosage, and scheduling [22]. Some studies reveal that cardiovascular complications after 5-FU administration usually happen during the first dose of chemotherapy. The mean time to the onset of the first cardiotoxicity symptoms is 12 h; however, some complications may occur up to 2 days after 5-FU infusion [25,26]. Another study shows that acute symptoms usually appear on the third or fourth day of a five-day continuous infusion and after the fourth intravenous administration in bolus treatment [27]. Table 1 presents the differences concerning the frequency of cardiotoxicity between various studies, which are possibly explained by the different populations and schedules included.

Cardiac muscle disturbances are one of the most common and most dangerous side effects of fluoropyrimidine treatment. Their occurrence should always be considered before the prescription of chemotherapy, as it may have severe consequences on a patient’s health. The differing frequency of cardiotoxicity events described in studies may result from the subtleness of symptoms and diagnostic difficulties.

## 3. Metabolism of 5-FU

Like some antineoplastic treatments, 5-FU is converted to highly cytotoxic metabolites, leading to both therapeutic and harmful consequences. The drug 5-fluorouracil can interfere with tumor cells due to its chemical structure. Some studies revealed the significance of fluorine derivatives in the human organism and emphasized the role of fluorine substitution, which may cause an increase in the molecule’s toxicity [38]. The van der Waal’s radius of the fluorine atom (1.35 Å) is virtually identical to that of hydrogen (1.2 Å) [39].

**Figure 1:**
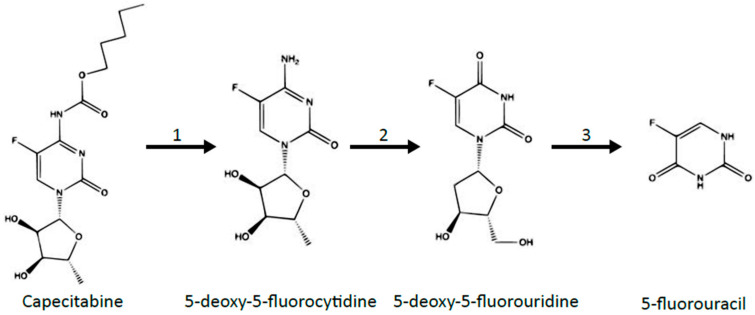
The three-step metabolism of capecitabine, another commonly used antineoplastic drug converted to 5-fluorouracil. 1—carboxylesterase; 2—cytidine deaminase; 3—thymidine phosphorylase. Reactions occur in the following sites: 1—liver; 2—liver and tumor; 3—tumor. Based on: -Martino et al. with modification.

**Figure 2:**
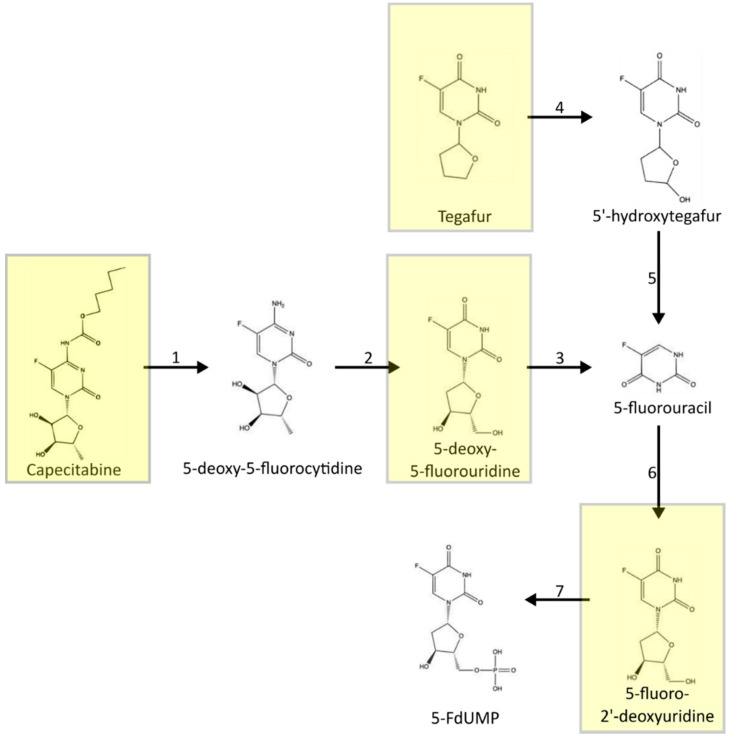
Metabolism of the prodrugs of 5-fluorouracil: capecitabine, 5-deoxy-5-fluorouridine, 5-fluoro-2′-deoxyuridine, and tegafur. 1—carboxylesterase; 2—cytidine deaminase; 3—thymidine phosphorylase; 4—cytochrome P450 enzymes; 5—spontaneous degradation; 6—thymidine phosphorylase; 7—thymidine kinase. dehydrogenase; 14-dihydropyrimidase; 15-β-ureidopropionase; 16- aconitase inhibition. Based on: Muhale et al. and Shirasaka et al. with modification.

**Figure 3:**
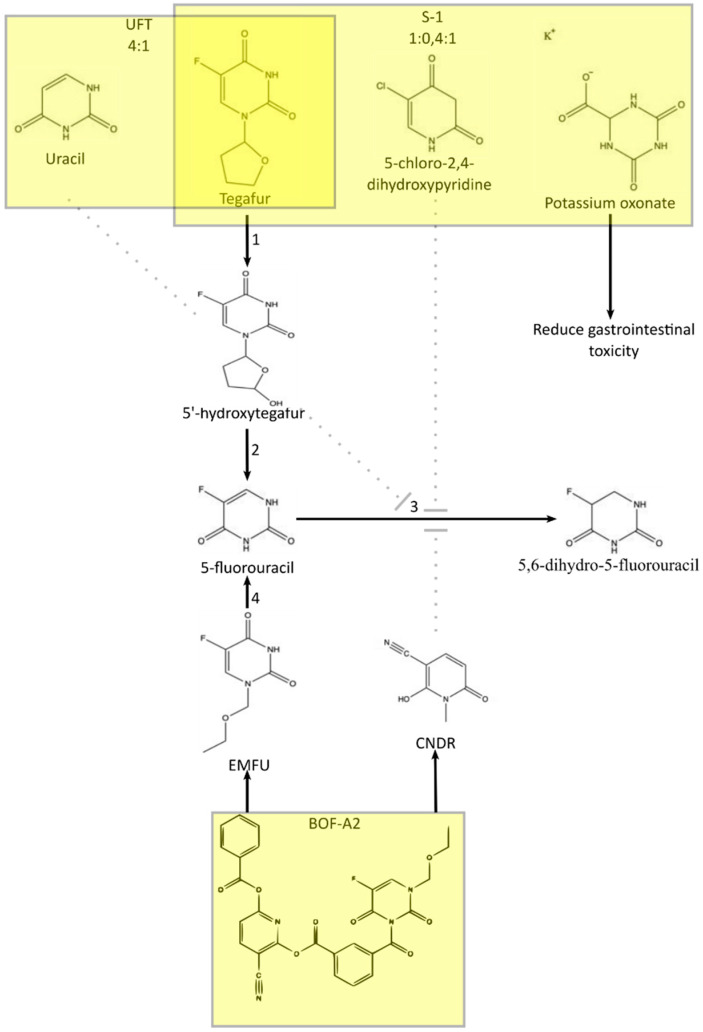
Metabolism of the prodrugs of 5-fluorouracil: UFT, S-1, and BOF-2A. 1—cytochrome P450 enzymes, 2—spontaneous degradation, 3—DPD, dihydropyrimidine dehydrogenase, and 4—hepatic microsomal enzymes. EMFU—2,6-dihydroxy-3-cyanopyridine; CNDP-3-cyano-2,6-dihydroxypyridine. Based on: Mallet–Martino et al., Miura et al., and Shirasaka et al. with modification.

Figure 1 presents the conversion of the oral prodrug capecitabine to 5-FU in three metabolic steps, with the last steps being catalyzed by thymidine phosphorylase [40]. It should be noted that capecitabine bioavailability can reach up to 100% [41]. Another similar chemotherapeutic agent is 5-deoxy-5-fluorouridine, which is converted to 5-FU by thymidine phosphorylase, as presented in Figure 2. The agent 5-fluoro-2′-deoxyuridine transformed into 5-fluorodeoxyuridine monophosphate by using thymidine kinase. Thus, 5-fluoro-2′-deoxyuridine administration causes inhibition of thymidylate synthase and disturbances in DNA structure. Tegafur is a prodrug metabolized into 5-fluorouracil by P450 cytochrome enzymes. A combination of antineoplastic drugs is available based on 5-fluorouracil, including UFT, S1, and BOF-2A, with their metabolism presented in Figure 3 [40,42,43,44]. UFT is a mix of tegafur, converted into 5-fluorouracil and uracil, which slows down the degradation of 5-FU. S1 contains tegafur, 5-chloro-2,4-dihydroxypyridine (which reduces 5-FU catabolism), and potassium oxonate, which is responsible for reducing gastrointestinal toxicity. BOF-2A is split into 2,6-dihydroxy-3-cyanopyridine and 3-cyano-2,6-dihydroxypyridine. The first one is converted into 5-fluorouracil by hepatic microsomal enzymes, and the second reduces 5-FU degradation.

**Figure 4:**
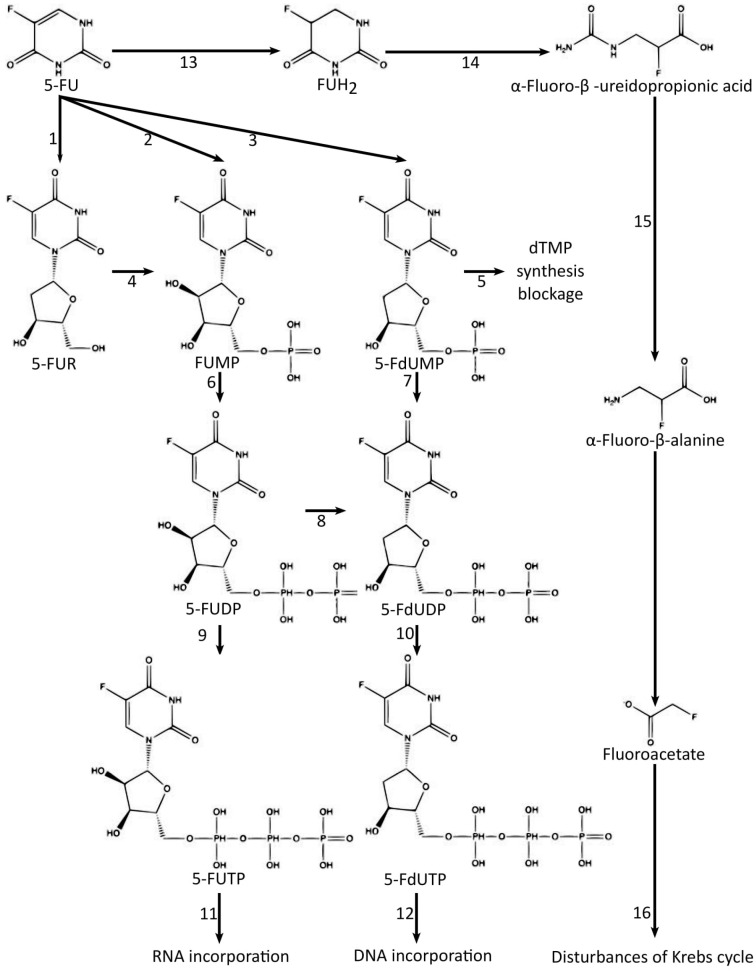
Metabolism of 5-fluorouracil. 1—UrdPase, uridine phosphorylase; 2—OPRT, orotate phosphoribosylotransferase; 3—dThdPase, thymidine phosphorylase and TK, thymidine kinase; 4—UK, uridine kinase; 5—thymidylate synthase; 6—uridine monophosphate kinase; 7—deoxyuridine monophosphate kinase; 8—ribonucleotide reductase; 9—uridine diphosphate kinase; 10—deoxyuridine diphosphate kinase; 11—RNA polymerase; 12—DNA polymerase; 13—DPD, dihydropyrimidine dehydrogenase; 14—DPS- dihydropyrimidase; 15—UPβ1-β-ureidopropionase; 16—aconitase inhibition. These are seen as 5-FU, 5-Fluorouracil; 5-FUR; 5-Fluorouridine; 5-FUMP, 5-fluorouridine monophosphate; 5-FdUMP, 5-fluorodeoxyuridine monophosphate; 5-FUDP, 5-fluorouridine diphosphate; 5-FdUDP, 5-fluorodeoxyuridine diphosphate; 5-FUTP, 5-fluorouridine triphosphate; 5-FdUTP, 5-fluorodeoxyuridine triphosphate; FUH2, 5,6-Dihydro-5-fluorouracil. Based on: Diasio et al., Miura et al., Peters et al., and Shirasaka et al. with modification.

The action of 5-FU has significant effects, especially during the S phase of the cell cycle, which is strictly associated with the activation of 5-fluorouracil and its conversion to both FdUMP and FUTP [39,45,46]. Subsequent steps are presented in Figure 4. By and large, FdUMP causes numerous abnormalities of the double-helical structure of DNA, while FUTP mainly leads to RNA dysfunction. It remains unclear which mechanism contributes to antineoplastic effects to a greater extent. However, about 80% of 5-FU is eliminated through reductive catabolism via dihydropyrimidine dehydrogenase (DPD) [47]. Lishke et al. highlighted that DPD triggers catabolic reactions and the generation of specific metabolites, such as fluoroacetate (FAC). This compound can lead to the inhibition of aconitase, which is one of the Krebs cycle enzymes, which in turn may severely impair mitochondrial metabolism [48]. Fraile et al. reported that the half-life of 5-FU is usually around 10 min [49].

FdUMP competitively binds to thymidylate synthase along with 5,10-methylenetetrahydrofolate and forms a ternary complex. As a result, methyltransferase is effectively inhibited [46,50]. The enzyme plays a crucial role in the proper function of DNA. FdUMP significantly impairs cell growth, whereas its derivative, FdUTP, directly incorporates into DNA in place of dTTP; therefore, it may cause innumerable DNA abnormalities. On the other hand, FUTP is analogically built into RNA to easily disrupt its delicate structure. 

## 4. Symptoms of Fluoropyrimidine Cardiotoxicity

The clinical presentation of fluoropyrimidine cardiotoxicity includes a wide variety of symptoms, including angina pectoris, myocardial infarction, cardiogenic shock, arrhythmias (atrial fibrillation, ventricular arrhythmias, atrioventricular block,), coronary vasospasm, and heart failure [4,10,36,51,52,53] The ESC described 5-FU as a drug that may lead to arrhythmias including bradycardia, atrioventricular block, atrial fibrillation, supraventricular tachycardias, ventricular tachycardia/fibrillation, or sudden cardiac death. Furthermore, 5-fluorouracil is also associated with peripheral arterial toxicity (including Raynaud’s phenomenon), and ischemic stroke diseases [22]. Table 2 presents the most significant adverse reactions after administration of 5-FU. Landmark studies on 5-FU cardiotoxicity were considered, with most indicating the numerous consequences of coronary vasospasm, such as ischemic changes or dysrhythmias, as the most crucial adverse reactions. The mechanism of 5-FU explains clinically manifested chest pain and heart palpitations. Some adverse reactions, such as myocardial infarction, heart failure, or hypotension, could be life-threatening. Some articles also provide divergent information about the mortality rate after the administration of 5-FU, which may be explained by different schedules, routes of administration, populations of patients, health professionals’ awareness, and patient knowledge.

Studies presented an increased duration of QT in patients after fluoropyrimidine treatment, which persists for up to 6 months after administration [30,33,54]. Fluoropyrimidine treatment leads to both tachycardia and bradycardia [29,34,55]. Rezkalla et al. reported that the frequency of asymptomatic ST changes during 5-FU infusion is up to 68% of patients [24]. On the other hand, the multicenter study conducted by Płońska–Gościniak et al. (2017) showed that chemotherapy with 5-FU or capecitabine in colorectal cancer patients did not affect the conduction system, LV structural parameters, and systolic function as measured by LVEF. However, chemotherapy with 5-FU or capecitabine in colorectal cancer patients may trigger subtle changes in myocardial performance, which are solely detectable by tissue Doppler echocardiography after 12 months [54]. Some case repots described a 5-FU induced myocarditis with a set of symptoms typical for myocarditis after the first course of 5-FU treatment [56,57]. Many studies described Takotsubo cardiomyopathy that developed after 5-FU chemotherapy [58,59,60]. Takotsubo cardiomyopathy, also known as apical ballooning syndrome (ABS), is primarily induced by stress and the release of catecholamines, resulting in transient myocardial abnormalities and hyperkinesis. The disease may be easily confused with coronary artery disease; however, it does not have a long-lasting impact on ventricular walls [60]. Moriyama et al. (2019) described the case of a 69-year-old patient with frequent paroxysms of atrial fibrillation (AF) during combination chemotherapy with 5-FU, which was sensitive to antianginal agents. Coronary angiography performed within the chemotherapeutic period demonstrated moderate stenosis in the right coronary artery (RCA). Severe spasm at the proximal portion of the atrial branch of the RCA was induced by the acetylcholine provocation test, suggesting that 5-FU might have predisposed vasospasm in the RCA, and the subsequent atrial ischemia could have led to AF [61]. Ray et al. (2020) presented a case of simultaneous cardiotoxicity and stroke-like neurotoxicity in a patient treated with the FOLFOX regimen. This study suggests that 5-FU-induced vasospasm in coronary arteries and cerebral vasculature is likely to cause simultaneous cardiac and neurological events. Similar observations were not reported previously in the medical literature [62]. 

Capecitabine may also lead to a cardiotoxicity similar to 5-FU-induced cardiotoxicity, including angina pectoris, arrhythmias, and dyspnea [30,31,33,63]. Saunders et al. described a patient who developed capecitabine-induced acute myopericarditis [64]. Another example is a rare case of Takotsubo cardiomyopathy after the administration of capecitabine described by Qasem et al. [65].

**Table 2:** Frequency of signs and symptoms after the administration of 5-FU. *—retrospective study; #—prospective study.

Article	Frequency of Sings and Symptoms
Jensen et al. (2006) * [31]	Angina: 3.9% Arrhythmias 0.4%
Rezkalla et al. (1989) # [24]	ECG changes: 68%
De Forni et al. (1992) # [66]	Angina pectoris: 4.9% Hypotension: 0.3%
Tsavaris et al. (2002) # [67]	ECG changes: 4% Arrhythmias: 2.3% Chest pain: 2.1% Myocardial infarction: 1.6% Palpitation: 1.4% Conductive abnormalities: 0.9% Malaise: 0.5% Loss of consciousness: 0.5%
Kosmas et al. (2008) # [30]	ECG changes: 4% Chest pain: 1.7% Palpitation: 1.1% Malaise: 0.6%
Koca et al. (2011) # [33]	ECG changes: 30.8% Palpitation: 23% Angina: 9.6% Dyspnea 7.6% Tachycardia: 5.6% Hypotension: 3.8% Hypertension: 1.9%
Peng et al. (2018) # [35]	Ischemic change: 19.9% Arrhythmia: 16.8% Heart failure: 2.6% Myocardial infraction: 1.0%
Dyhl-Polk et al. (2020) # [37]	Acute coronary syndrome: 4% Chest pain: 0.7%

In summary, the average mortality rate due to fluoropyrimidines cardiotoxicity oscillates from 1.6% to 10.2%. Although oral capecitabine may also pose a risk of cardiovascular complications, its administration is sometimes more convenient for patients [9,10,11].

## 5. Routes of 5-FU Administration

Over the years, several routes of 5-fluorouracil administration have been explored. Nowadays, continuous infusion is of great significance; however, in some cases, intravenous bolus administration can be applied as well [68,69]. Other routes of 5-FU administration will also be considered. 

Continuous infusion ensures prolonged exposure, and thus the concentration of the drug is relatively steady and less harmful for most cells. Studies established that infusion may result in less toxic effects on the bone marrow or digestive system [70,71]. De Gramont et al. highlighted that 5-fluorouracil should be given in continuous infusion to increase efficacy [72]. Researchers observed that, after continuous infusion, troponins were visibly elevated, and the risk of cardiotoxicity was increased to 7–18% compared to intravenous bolus [73,74]. Unfortunately, various studies proved that a single bolus injection is not as effective as 5-FU continuous infusion. Shorter exposure to 5-FU does not prevent most tumor cells from further proliferating.

Some studies reported that intravenous bolus administration could be given from 7.2 to 17 mg/kg [75,76,77]. The 5-FU plasma half-life was estimated at around 7–28 min. Moreover, Heggie et al. indicated that the half-life of 5-FU catabolites could be much longer, lasting even up to 33 h [78].

The oral administration route was not proven effective enough, as the drug when taken orally may lead to unpredictable plasma concentrations due to its pharmacokinetics [49]. Bioavailability varies considerably between individuals, and thus a wide variety of adverse symptoms, including cardiotoxicity effects, can occur.

Pericardial infusion was reported by Lerner-Tung et al., by which 5-FU can reach high levels in the pericardial sac. Moreover, its half-life is thereby significantly increased compared to systemic administration (168 min vs. 7–28 min). Pericardial infusion was also proven to cause less harm to proliferating cells [79].

**Figure 5:**
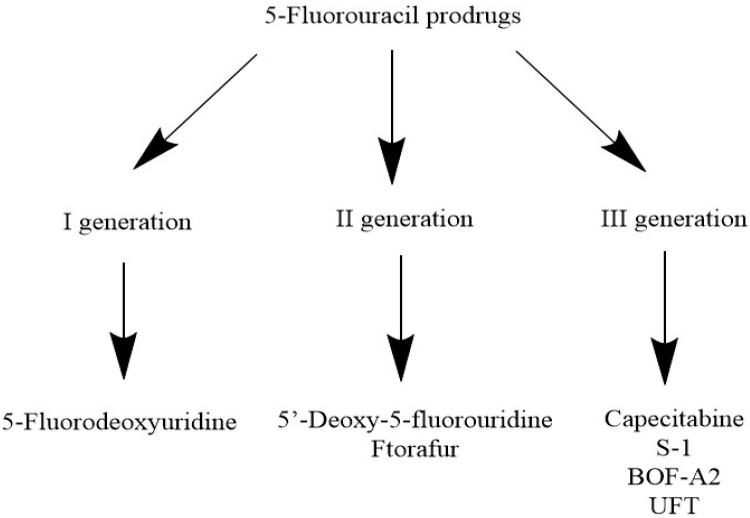
Classification of 5-fluorouracil prodrugs. Based on: Malet-Martino et al. with modification.

Although 5-FU results in effective chemotherapy, the intravenous route of administration remains relatively inconvenient for the patient requiring frequent infusion appointments and potentially risky catheterization [80]. Thus, several oral prodrugs of 5-FU have been developed, whose classification is presented in Figure 5. Capecitabine is presumably the most common and widely accepted 5-FU due to its convenient pharmacokinetics. The drug can be administered orally and is converted to 5-FU through three activation steps, which translates to less severe systemic toxicities. Nowadays, capecitabine is preferably used in metastatic colorectal cancer [20]. Other 5-FU prodrugs are 5-fluorodeoxyuridine, 5′-deoxy-5-fluorouridine, BOF-A2, ftorafur, UFT, and S-1 [40,42,44]. The 5-fluorodeoxyuridine is a first-generation prodrug, while 5′-deoxy-5-fluorouridine and ftorafur are second generation prodrugs; however, they are not commonly used in everyday practice. The third generation includes capecitabine, mentioned above, and DPD inhibitors (BOF-A2, UFT, and S-1), which prevent the enzymatic degradation of 5-FU. For example, BOF-A2 contains both 3-cyano-2,6-dihydroxy pyridine (CNDP), a competitive inhibitor of DPD, and 1-ethoxymethyl-5-fluorouracil (EMFU). Another 5-FU prodrug, UFT, has been extensively examined and applied in the treatment of many tumors, such as breast, colon, and stomach with good tolerance. Finally, S-1 was developed in 1996 to overcome burdensome side reactions, but the drug did not show greater efficacy than other prodrugs. 

## 6. Mechanisms of Fluoropyrimidines Cardiotoxicity

Using 5-FU treatment may lead to myocardial ischemia and coronary artery disease, including Prinzmetal’s angina, heart failure, and arrhythmic changes [81], yet detailed mechanisms of cardiotoxicity induced by fluoropyrimidines remain unclear and uncertain, but are definitely multifactorial [7,82,83,84]. The European Society of Cardiology identified coronary vasospasm and endothelial injury as key pathophysiologic processes [22]. An endothelial-dependent vasospasm is related to direct endothelial dysfunction, whereas an endothelial-independent vasospasm refers to primary smooth muscle dysfunction [7]. Based on preclinical studies, endothelial dysfunction should activate apoptosis and autophagy pathways in both endothelial cells and myocytes [85,86]. Another consequence of endothelial damage is an increased blood level of vasoconstrictors, such as endothelin-1 and urotensin-2, which was observed in 5-FU treated patients [87,88]. Severe endothelial damage, together with platelet accumulation and fibrin formation, was also observed in 5-FU-treated rabbits in scanning electron microscopy studies [89]. Thus, after vessel injury, a thrombogenic effect may also be triggered. The pro-coagulant effect is further enhanced due to the primary cause of the patients’ treatment, i.e., tumors [90,91]. Moreover, due to endothelial dysfunction, or eNOS abnormalities, acetylcholine can cause paradoxical vasoconstriction instead of vasodilation [92,93]. In this way, chronic vasoconstriction related to 5-FU treatment might have cardiotoxic consequences. Primary smooth muscle dysfunction should result in vasoconstriction in the presence of a functionally intact endothelium. Such contraction of vascular smooth muscles was proven in vitro when aortic rings of white rabbits were exposed to increasing doses of 5-FU. This endothelium-independent vasoconstriction was mediated by the activation of kinase C (PK-C) in vitro [51,94]. It is worth mentioning that Salepci et al. demonstrated that 5-FU-induced coronary vasospasm was independent of angiotensin II levels in 31 patients treated with 5-FU/leucovorin [95].

Using 5-FU treatment is also associated with enhanced oxidative stress due to the formation of reactive oxygen species, lipid peroxidation, and the decrease in glutathione level, with cardiomyocytes being especially vulnerable to reactive oxygen species damage because of their numerous mitochondria [7,82,83,84,96]. For example, Durak et al. demonstrated that the administration of 5-FU to guinea pigs reduced the activity of superoxide dismutase and glutathione peroxidase with a concomitant increase in catalase activity and concentration of malondialdehyde [97]. Similarly, an increase of the oxidative stress was observed in vitro in 5-FU treated cardiomyocytes [86]. Moreover, animal studies revealed the significant role of 5-FU degradation to highly toxic metabolites, which could interfere with the Krebs cycle [98,99]. In a single case report, an increased serum level of alpha-fluoro-beta-alanine, a precursor of fluoroacetate, was reported in a patient who received a continuous intravenous infusion of 5-FU and experienced precordial pain with right bundle branch block [100].

Furthermore, 5-FU-induced cardiotoxicity could be related to the disruption of the energetic metabolism of erythrocytes, which was observed in both in vivo and in vitro studies [101,102]. A rapid increase in O2 consumption leads to severe changes in the metabolism of phosphate compounds in erythrocytes, while a drastic decrease in ATP levels causes disruptions in their structure and functioning, such as irreversible echinocytosis or increased membrane fluidity, diminishing their ability to deliver oxygen. As a result, it makes oxygen transport or delivery more difficult, leaving metabolically active organs like the heart with insufficient oxygen supply, and inevitably resulting in ischemic damage [101,102].

**Figure 6:**
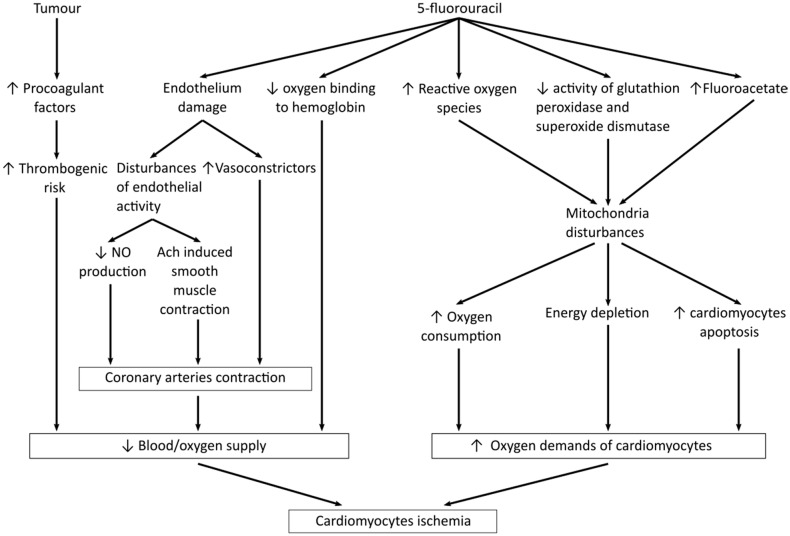
Mechanisms leading to cardiotoxicity.

Figure 6 summarizes the possible mechanisms of 5-FU-induced cardiotoxicity, which are mostly based on preclinical studies only. We have yet to discover the mechanistic relationships that would let us predict the chances of serious adverse cardiac reactions in patients treated with fluoropyrimidines, and we have also yet to learn to react effectively enough to avoid those complications.

## 7. Diagnostics of 5-FU Cardiotoxicity

The most widely used technique to monitor cardiotoxic effects is electrocardiography. Indeed, patients treated with fluoropyrimidines present a wide range of electrocardiographic changes, such as increased P duration, P dispersion, QT interval, QT dispersion, T wave inversion, and ST elevation [29,30,33,103]. Koca et al. reported new ECG abnormalities in 33% of patients after capecitabine treatment, the most common being sinus tachycardia and a prolonged QTc interval [33]. Rezkalla et al. described asymptomatic ST-segment elevation in 24% of patients before 5-FU administration and in 68% while they were being treated [24]. As far as electrophysiological changes are concerned, it has been noted that various arrhythmias can occur, including premature ectopic beats, atrioventricular block, atrial fibrillation, and ventricular extrasystoles [30,33].

Heart rate variability (HRV) may be an early indicator of anthracycline-induced cardiotoxicity, but there is a lack of evidence in using HRV as an indicator of 5-FU cardiotoxicity [104].

Elevation of cardiac troponins was noted by Holubec et al. in almost 57% of patients, while other researchers reported no significant increase after fluoropyrimidine treatment [74,95,103]. Brain natriuretic peptide (BNP) elevation was observed in 29–48% of patients [32,74]. Studies claimed no significant increase in creatine kinase (CK) while retrospective study (Saif et al. 2009) reported increased CK or lactic dehydrogenase (LDH) in 12% of patients [69,103,105,106].

Unfortunately, there are discrepancies between studies in echocardiographic results. Some studies reported no differences in echocardiography, while other investigators observed significant changes after fluoropyrimidine treatment [54,66,103,105,107].

Polk et al. noted that the evaluation of histopathological changes is still difficult, as myocardial biopsy samples are hardly accessible [83].

Various diagnostic procedures were examined to assess their relevance in predicting 5-FU-related cardiotoxicity. For the time being, the most essential for early diagnosis seems to be electrocardiographic monitoring and routine clinical observations of patients and their reported symptoms. 

## 8. Cardiotoxicity Risk Factors

In 2020, the European Society for Medical Oncology emphasized that patients with a pre-existing cardiovascular disease or cardiovascular risk factors are at greater risk of oncology treatment-induced cardiotoxicity. Recommendations suggest screening and treatment of cardiovascular risk factors. They also noted the important role of close and early collaboration between cardiologists, oncologists, hematologists, and radiation oncologists [108]. In 2020, the American Society of Clinical Oncology (ASCO) also suggested assessment and treatment of cardiovascular risk factors before initiation of chemotherapy [23].

A clinical history of cardiac diseases was noted as a relevant risk factor for cardiotoxicity by Meyer et al. Their work presented that patients who were suffering from ischemic heart disease had a relative risk of 8 for complications compared with those without ischemic heart disease [28]. Labianca et al. observed that pre-existing ischemic heart disease increased the risk of cardiotoxicity by 1.1% to 4.5% [10]. Jin et al. included pre-existing cardiac disease as the risk factor after 5-FU administration [36]. The results of the study published by Polk et al. in 2016 suggested that cardiac comorbidity was the risk factor for symptomatic cardiotoxicity and increased risk of 3.7% to 16.7% [63]. Nonetheless, further research is needed to check whether smoking cessation can prevent some cases of symptomatic cardiotoxicity. However, another review written by Depetris et al. showed the lack of solid evidence for the occurrence of the 5-fluorouracil-induced cardiotoxicity among patients with a history of cardiac disease or the presence of cardiovascular risk factors [82].

Researchers reported that the risk of cardiac complications during 5-FU-based chemotherapy was higher in renal insufficiency, cardiac comorbidities, or more extended therapies [31,73]. Moreover, the analysis performed by Jin et al. presented the significant correlation between the incidence of cardiotoxicity in all patients and their age, smoking history, ECOG PS >2, anemia (hemoglobin <90 g/L), ≥3-grade neutropenia, or respiratory infections [36]. Meyer et al. presented in a prospective study that receiving nitrates and calcium channel blockers increases the risk of cardiovascular complications [28]. 

Robben et al. (1993) concluded that polychemotherapy, including platinum compounds, was a probable cause of increased cardiotoxicity [34,109]. Then, the pooled analysis performed by Abdel-Rahman reported that bevacizumab-containing therapies could be associated with an increased risk of ischemic events, and panitumumab-containing therapies could cause a higher risk of arrhythmias in comparison to other 5-FU based regimens [110]. Kelly et al. indicated raltitrexed as a good option for vulnerable patients to avoid significant cardiac risk factors during 5-FU therapy. It is a folate analogue with activity concentrated on the inhibition thymidylate synthase. Raltitrexed is advantageous in palliative care and the treatment of advanced rectal cancer, where the 5-FU and folinic acid-based therapies are not appropriate or tolerated. Moreover, raltitrexed is a relevant option for patients with metastatic colorectal cancer and cardiovascular risk factors [111,112,113,114].

Another risk factor is prior or concurrent radiation chest therapy. Concededly, some authors reported a lack of association between a history of chest radiation and increased cardiac risk during 5-FU-based chemotherapy [28]. Nevertheless, recent data classified the history of prior or active chest wall radiation therapy among patients treated with fluoropyrimidines as a relevant risk factor. These observations emphasize the need for complex clinical care and heightened awareness for this group of patients [16,34]. Chemoradiotherapy incorporating 5-FU significantly increases the risk of congestive heart failure compared to radiotherapy without any chemotherapeutic drugs [115].

Some authors suggest that individual susceptibility to cardiotoxic side effects of 5-FU administration corresponds with different enzyme polymorphisms in the catabolizing pathways rather than pre-existing cardiovascular comorbidities [116]. For example, Ma et al. mentioned DPD (dihydropyrimidine dehydrogenase) as one such enzyme affecting susceptibility to cardiotoxicity, whose deficiency was noted among 3–5% of 5-FU patients, and it resulted in a lower ability to degrade 5-FU [117]. Milano et al. (1999) published a study in which they analyzed the correlation between dihydropyrimidine dehydrogenase deficiency and 5-FU-related toxicity. Their conclusions suggested that clinically manifested overexposure to 5-FU seemed to be linked to DPD deficiency. A similar significant relationship between lymphocytic DPD activity and 5-FU clearance has been previously reported [116,118]. It should be noted that genetic predisposition plays a significant role in cardiovascular problems after 5-FU therapy. 

In one study involving a group of patients with a variant in the gene promoter of the thymidylate synthase, the concentration of this enzyme was significantly increased. Moreover, these patients presented inadequate response to treatment, as well as fewer adverse effects [119,120]. One case report referred to a patient with coronary vasoconstriction and confirmed variant in the DPD gene [25,121]. Likewise, a high risk of the rapid onset of severe toxicities is observed in the case of variants in OPRT (orotate phosphoribosyltransferase), with an average incidence of 16% to 17% in the United States [117,122]. This variant is related to an increase in 5-FU anabolism to form toxic intracellular 5-fluorouridine nucleotides. Other variants associated with the excessive fluoropyrimidine toxicity include variants of TYMS (thymidylate synthase) and MTHFR (methylene tetrahydrofolate reductase) [123,124]. Recently, Saif et al. (2016) reported the case of a patient diagnosed with Takotsubo cardiomyopathy, secondary to 5-FU therapy. The concomitant presence of two pharmacogenetic abnormalities of TYMS and DPYD was unique to this patient, and that combination of variants has never been reported in medical literature before [125].

The results presented by Milano et al. (1999) showed that the overwhelming majority of cases of DPD deficiency was in women (79%). A sex-related tumor profile cannot explain this observation because most cases were digestive tract cancers. This work showed that female patients were prone to carrying a DPD-deficiency syndrome, and they should be given priority in systematic investigations of DPD activity before 5-FU-based chemotherapy [116]. On the other hand, Raber et al. (2020) published the results of a retrospective case-control study that found that men were highly represented in cases of cardiotoxicity; however, prior observations suggested that the tendency to adverse cardiac effects after 5-FU therapy is more common among female patients [126]. An ASCO study also claimed health disparities between racial and ethnic groups. For example, African Americans are more likely to suffer from hypertension, diabetes, and cardiovascular diseases. Moreover, African American women with breast cancer have poorer survival rates when compared with nonminority groups [23].

Nevertheless, adverse cardiac effects associated with 5-FU-based chemotherapy were sometimes observed among the patients with no known risk factors [127]. That is why robust cardiac management during chemotherapy is recommended. It should consist of a cardiac screening test to estimate the risk of cardiotoxicity in individual cases, reduction of anemia before treatment initiation to improve cardiac tolerance, and observation of bone marrow suppression to adjust doses of 5-FU according to the guidelines for chemotherapy regimens [36]. Some studies recommend that when the diagnosis of fluoropyrimidine cardiotoxicity is unclear, testing for genetic variants affecting thymidylate synthase and dihydropyrimidine dehydrogenase activity may help support the diagnosis [126,128,129].

Since patients who undergo 5-FU continuous infusion are particularly prone to cardiac symptoms, they should avoid excessive physical activity. It is worth mentioning that effort-induced myocardial ischemia (EMI) frequently occurs during 5-FU administration [21].

Even though many studies have been conducted and many factors were assessed, there is still a lack of conclusions as to the clear triggers of 5-FU related cardiotoxicity. For example, factors such as sex or history of cardiac diseases may be correlated with a higher rate of side effects. However, severe clinical manifestations of cardiotoxicity were also described among patients with no history of cardiac disorders. Furthermore, some studies also demonstrated that the risk of cardiac side effects was higher due to earlier renal insufficiency, coronary artery disease, heart failure, or more extended therapies. On the other hand, some authors suggest that the susceptibility to adverse reactions corresponds more to different enzyme polymorphisms in the catabolizing pathways than to pre-existing cardiovascular comorbidities. Thus, it is still challenging to put forth overall conclusions, and every patient should be managed individually.

## 9. Management of Fluoropyrimidine Cardiotoxicity

Saif et al. described that even though 92% of patients with an episode of 5-FU-related cardiotoxicity survived and recovered, the noted mortality rates were still high [69]. Thalambedu et al. summarized that in case of any signs of 5-FU cardiotoxicity, therapy should be discontinued and replaced by anti-anginal agents. Some studies suggested that this intervention could bring about the disappearance of cardiac symptoms in as much as 69% of patients [130].

First, in patients with acute chest pain, which is the most common manifestation of 5-FU cardiotoxicity, a precise anamnesis needs to be taken and a cardio-pulmonary physical examination conducted, including an assessment of cardiac risk factors and details of the chemotherapy protocol such as dosage, routes of administration, and the date of the last cycle before the onset of symptoms. Moreover, non-invasive tests, including ECG, need to be conducted to check for any signs of ischemic ST changes or arrhythmias. Echocardiographic examination, cardiac troponins, BNP levels, and CT coronary angiography should be performed to establish the diagnosis. The necessity of constant monitoring of cardiac biomarker levels among patients who undergo 5-FU-based chemotherapy remains undetermined. However, the 2012 Clinical Practice Guidelines of the European Society of Medical Oncology recommend it in the case of patients with a history of cardiovascular diseases as a class III/IV recommendation [131,132]. The results of non-invasive tests may indicate the need for invasive tests, such as a coronary angiogram, which are generally dedicated for patients with known risk factors for cardiovascular disease [7]. The American College of Cardiology/American Heart Association suggests that urgent coronary angiography should be performed in ACS (acute coronary syndrome) or the need to exclude ACS. Invasive pharmacologic provocation during coronary angiography seems to be a practical test to diagnose functional coronary abnormalities. Nonetheless, this method is not widely available, and its usefulness in risk-stratification in the case of 5-FU cardiotoxicity remains unclear, necessitating further studies [133].

The next step in managing patients with clinical manifestation of anginal chest pain should be nitrates, beta-blockers, and calcium channel blockers. These medications are standard initial therapy despite the debate on their efficacy [25,30,31,67,134]. Steger et al. stated that even though some studies could not confirm the effect of calcium channel blockers or nitrates in reducing the risk of cardiotoxicity, prophylactic administration is widespread [135]. It is also worth mentioning that the direct toxic effect of 5-FU on the vascular endothelium may cause severe thrombogenic disorders. This problem was addressed by Kinhult et al., and their in vivo experiment on rabbits suggested a protective dalteparin treatment. However, no such clinical trials have ever been performed [89]. Sara et al. (2018) summarized that management of patients with cardiac adverse effects after treatment with 5-FU should be focused on determining whether 5-FU can be attributed to the cardiotoxicity and identifying and treating other coexisting coronary disease. Moreover, it is crucial to determine whether further 5-FU is required, or if any acceptable alternative treatment can be safely considered. When further doses of 5-FU are required, clinicians should continue cautiously, consider using prophylactic antianginal therapy, and monitor patients closely with a low threshold to terminating therapy. However, to clarify the optimal strategy, randomized clinical trials comparing different approaches to manage these patients will be essential [7]. 

Ma et al. highlighted the use of uridine triacetate as a highly effective and safe antidote in the case of 5-FU-induced vasospasm. In their study, 96% of patients who overdosed 5-FU, and received uridine triacetate, survived. On the contrary, the survival rate for similar patients who did not receive the antidote was only 16%. The action of uridine triacetate is associated with competitive inhibition of cytotoxic components responsible for incorporating 5-FU into RNA. The initiation of treatment within 96 h after termination of 5-FU infusion was noted as a significant factor in survival. These findings illustrate the potential severity of early-onset toxicities and the need for immediate recognition and treatment. Nevertheless, prospective trials designed for patients with FIC (fluoropyrimidine-induced cardiotoxicity) need to be performed to investigate and describe the accurate role of this drug in treatment [117].

Many studies reported the negative impact of hydrogen peroxide on cultured cardiac myocytes. According to Lin et al., a compound can protect cardiac cells against cytotoxic H2O2. Non-mitogenic human acidic FGF has been proven to be cardioprotective; however, its effect during 5-FU therapy is still unknown [136]. Probucol is an antioxidant which can also reduce lipid levels. The drug was tested to prevent endothelial damage of the central artery in rabbit ears after 5-FU therapy. The probucol-treated animals were significantly less affected by 5-FU therapy, and their results were similar to the control group. Clotting and endothelial injury were observed only in the group treated with 5-FU. This study revealed the cardioprotective function of probucol during 5-FU administration [137]. Another study indicated a significant role of clenbuterol, which increases Ca2+-ATPase activity and reduces oxidative stress. Moreover, the compound prevents cardiac cells from programmed cell death, and it may improve ventricular diastolic function. Clenbuterol is a crucial cardioprotective factor in the presence of H2O2 in vitro; however, its role in vivo, especially during chemotherapy, is unclear [138]. The research conducted by Sun et al. showed that propofol conditioning might also protect against cardiomyocyte hypoxia and myocardial ischemia. This phenomenon was accompanied by increased endocannabinoid (AEA and 2-AG) release and alleviated oxidative stress [139].

Other unmissable findings were reported by Altieri et al. They performed a study to assess the role of glucagon-like-peptide 1 in counteraction of 5-FU-induced endothelial cell senescence and reduction of eNOS as well as SIRT-1 expression. GLP-1 analogs and degradation inhibitors are clinically available and were reported to exert beneficial cardiovascular actions. Likewise, their research presented that GLP-1 prevented endothelial senescence due to 5-FU and treated its vascular toxicity in vitro. However, further studies are necessary to check whether similar results may be observed in vivo and in the clinical scenario [140].

Zhang et al. described the cardioprotective action of coenzyme complex in combination with chemotherapy administered to older adults with gastrointestinal cancer. This substance decreases cardiotoxicity by enhancement of mitochondrial energy production, cell membrane stabilization, and beneficial effects on the metabolism of long-chain fatty acids. More data need to be collected to verify and confirm the clinical use of the coenzyme complex [141]. Moreover, it is described that calcitriol has a protective effect on organ damage after 5-FU administration; however, the function of the substance remains unclear [142].

Bi et al. (2018) published the results of their work, which indicated indole alkaloid derivative B (IADB) as a mitigating agent against 5-FU-induced cardiotoxicity. Researchers examined its antioxidant activation as well as modulation of autophagy. The administration of bifunctional IADB may be beneficial, especially during high-dose chemotherapy, as it can reduce cardiotoxicity by preventing reactive oxygen species overproduction. Further investigation is required, and other substances are also being investigated [143].

Rateesh et al. (2015) reported the successful use of peripheral venoarterial extracorporeal membrane oxygenation (ECMO) in a patient with acute 5-fluorouracil–induced cardiomyopathy. Together with medical therapy for heart failure, almost complete recovery of cardiac function was possible. The authors emphasized that early use of ECMO for hemodynamic stabilization enables myocardial recovery after improving initial stunning. An aggressive strategy, including mechanical support, has been associated with improved survival compared with conservative treatment [144]. In 2017, Sundaravel et al. reported the first case of FOLFOX-induced Takotsubo cardiomyopathy treated with an Impella device. Their patient had severe cardiogenic shock with an EF of 20%, which improved significantly after three days of using the Impella Assist Device [58].

To conclude, different ways of management in the case of 5-FU induced cardiotoxicity are proposed, but most of them depend on the individual state of the patient. The general rule worth remembering is discontinuing chemotherapy and replacing it with anti-anginal drugs after any symptom of cardiotoxicity. A precise anamnesis and a cardio-pulmonary physical examination needs to be taken, including assessing cardiac risk factors and the details of the chemotherapy such as dosage, routes of administration, and the date of the last course before symptoms occurred. Moreover, non-invasive tests, including ECG, need to be conducted to check for any signs of ischemic ST changes or arrhythmias. Finally, despite the debate about its efficacy, standard initial therapy with nitrates and calcium channel blockers should be initiated. Any further clinical decisions should be based on the patient’s clinical state and other possible therapeutic options.

## 10. Reintroduction of 5-FU

The reintroduction of 5-FU in patients with a previous history of cardiotoxic events after 5-FU administration is not currently recommended. It was documented that repeated exposure to 5-FU can cause a recurrence of cardiotoxicity in 82–100% of patients, with a death rate in these cases approaching 18% [26,69]. After the resolution of cardiotoxic symptoms, rechallenge may be considered if it is the only therapeutic option to improve the patient’s chances for survival. It should be preceded by a multidisciplinary discussion between oncologists and cardiologists. In addition, risk stratification, coronary evaluation, and treatment following ACA/AHA guidelines need to be performed. Patients with antecedent CAD (coronary artery disease) are recommended to reduce other risk factors, including smoking, hypertension, or uncontrolled diabetes [16]. 

Moreover, a reduction in the dosage of 5-FU seems to be a viable option, and even though we still have no confirmation of the efficacy of the pharmacologic prophylaxis, a prolonged course of pretreatment with nitrates and calcium channel blockers and its continuation during drug infusion has been proposed. To mitigate the risk, patients should also be provided with careful cardiac monitoring, and the suggested method for drug administration is a bolus regimen that has been proven to be safer than a continuous infusion of 5-FU [30,134,145,146]. The study performed by Jensen et al. showed that a combination of prophylaxis with dose-reduced 5-FU decreased the risk of cardiotoxicity in 9 out of 12 patients [31]. Some authors tried to find indications for other prophylaxis agents. For example, Salepci et al. performed a study to assess the prophylactic use of ACEI. They measured the level of angiotensin II among the patients treated with 5-FU bolus administration. However, no changes were observed compared to the control group, and their theory was disproven [95].

Some case reports described successful conversion from 5-FU to capecitabine without a recurrence of cardiac symptoms. They show that capecitabine may be an option for patients who develop 5-FU-induced chest pain. Nevertheless, these patients should be very closely monitored for the recurrence of symptoms [147,148]. The European Society of Cardiology prepared dedicated guidelines about the cardiotoxicity of oncology treatment. The authors emphasized that the final decision about treatment continuation or cessation has to be made by responsible physicians based on the individual characteristics of the patient [22].

Nevertheless, when the completion of the fluoropyrimidine-based regimens is limited, alternative strategies should be considered. An example of a safe therapeutic option in patients who experienced FIC after prior 5-FU infusion seems to be raltitrexed [111,114].

Another possible alternative is TAS-102 (trifluridine/tipiracil). This is a combination of substances that contain DPD inhibitors. As a result, less FBAL (α-fluoro-β-alanine) metabolite is concentrated, and lower rates of cardiac complications may be observed [149,150]. TAS-102 is an oral fluoropyrimidine, and its use was proposed in the recent review written by Petrelli et al. They analyzed the incidence of cardiotoxic effects in phase I, phase II, and phase III trials. The observations included three cases of cardiac events. Based on these data and different pharmacokinetics of TAS-102, they suggested this drug as an alternative, especially among patients at increased cardiovascular risk [151].

All in all, the reintroduction of 5-FU after an adverse cardiac reaction is not recommended. Instead, rechallenge may be considered only after resolving cardiotoxic symptoms and if it is the only therapeutic option to improve the patient’s chances for survival. This decision requires aggressive inpatient supportive care, constant cardiac monitoring, and preventative strategies, including decreasing the 5-FU dose and vasodilator therapy. However, when the completion of the fluoropyrimidine-based regimen is limited, clinicians should consider other, less cardiotoxic alternatives and ways of management. For the time being, the therapeutic options that seem safe enough include raltitrexed and TAS-102.

## 11. Summary

Cardiovascular disease and cancer are the two leading causes of death worldwide. Overall, cancer morbidity and survivorship are increasing, yet fluoropyrimidine induced cardiotoxicity is a growing problem because they are commonly used antineoplastic agents. Pathogenesis of the fluoropyrimidine induced cardiotoxicity remains unclear. A multifactorial mechanism is considered the most comprehensive, including endothelial damage, thrombogenicity, oxidative stress, mitochondrial disturbances, direct damage, and hemoglobin defects. Most commonly, patients suffer from heart ischemia or arrhythmias, which while reversible may nevertheless be fatal. Currently, cessation of fluoropyrimidines and treatment with antianginal agents such as nitrates, beta-blockers, and calcium-blockers, despite discrepancies about their efficiency, is recommended in case of fluoropyrimidine-induced cardiotoxicity. Reintroducing fluoropyrimidine is not recommended unless they significantly improve prognosis after profound cardiovascular assessment and implementation of prophylaxis. Using alternative antineoplastic agents should be considered, including raltitrexed or S-1. A well-designed human study with throughout risk factor evaluation, repeated clinical observation, electrocardiographic and biomarkers assessment may potentially explain existing discrepancies among studies related to fluoropyrimidine induced cardiotoxicity, and they could possibly facilitate preventive measures.

## Figures and Tables

**Table 1:** Frequency of cardiotoxicity after the administration of fluoropyrimidines. *—retrospective study; #—prospective study.

Article	Frequency of Cardiotoxicity
Meyer et al. (1997) # [28]	5-FU-related: 1.9%
Wacker et al. (2003) # [29]	5-FU-related: 19%
Kosmas et al. (2006) # [30]	Fluoropyrimidines-related: 4.0%
Jensen et al. (2006) * [31]	5-FU-related: 5.9% Capecitabine-related: 2.8%
Jensen et al. (2010) # [32]	5-FU-related: 8.5%
Koca et al. (2011) # [33]	Capecitabine-related: 34.6%
Khan et al. (2012) * [34]	5-FU-related: 19.9%
Lestuzzi et al. (2014) # [21]	5-FU-related: 5.9%
Peng et al. (2018) # [35]	5-FU-related: 25% Capecitabine-related: 33.8%
Jin et al. (2019) * [36]	5-FU-related: 29.5%
Dyhl-Polk et al. (2020) * [37]	Fluoropyrimidines-related: 5.2%

## Data Availability

Not applicable.
